# New strategies to improve clinical outcomes for diabetic kidney disease

**DOI:** 10.1186/s12916-022-02539-2

**Published:** 2022-10-10

**Authors:** Thomas Forst, Chantal Mathieu, Francesco Giorgino, David C. Wheeler, Nikolaos Papanas, Roland E. Schmieder, Atef Halabi, Oliver Schnell, Marina Streckbein, Katherine R. Tuttle

**Affiliations:** 1 Clinical Research Services, Mannheim GmbH, Grenadierstrasse 1, D-68167 Mannheim, Germany; 2 Department of Endocrinology, UZ Gasthuisberg, Katholieke Universiteit, Leuven, Belgium; 3 Department of Emergency and Organ Transplantation Section of Internal Medicine, Endocrinology, Andrology and Metabolic Diseases, University of Bari Aldo Moro, Bari, Italy; 4 Department of Renal Medicine, University College London, London, UK; 5 Diabetes Centre, Second Department of Internal Medicine, Democritus University of Thrace, Alexandroupolis, Greece; 6 Department of Nephrology and Hypertension, University Hospital Erlangen, Erlangen, Germany; 7 Clinical Research Services, Kiel, Germany; 8 Forschergruppe Diabetes e.V., Munich, Germany; 9 Division of Nephrology, Institute of Translational Health Sciences, University of Washington, Seattle, WA USA

**Keywords:** Type 2 diabetes, Diabetic kidney disease, Kidney protective agents

## Abstract

**Background:**

Diabetic kidney disease (DKD), the most common cause of kidney failure and end-stage kidney disease worldwide, will develop in almost half of all people with type 2 diabetes. With the incidence of type 2 diabetes continuing to increase, early detection and management of DKD is of great clinical importance.

**Main body:**

This review provides a comprehensive clinical update for DKD in people with type 2 diabetes, with a special focus on new treatment modalities. The traditional strategies for prevention and treatment of DKD, i.e., glycemic control and blood pressure management, have only modest effects on minimizing glomerular filtration rate decline or progression to end-stage kidney disease. While cardiovascular outcome trials of SGLT-2i show a positive effect of SGLT-2i on several kidney disease-related endpoints, the effect of GLP-1 RA on kidney-disease endpoints other than reduced albuminuria remain to be established. Non-steroidal mineralocorticoid receptor antagonists also evoke cardiovascular and kidney protective effects.

**Conclusion:**

With these new agents and the promise of additional agents under clinical development, clinicians will be more able to personalize treatment of DKD in patients with type 2 diabetes.

## Background

According to the International Diabetes Federation, 537 million adults (20–79 years of age) were living with diabetes mellitus worldwide in 2021, and this number is expected to increase to more than 780 million by the year 2045 [1]. Of these, an estimated 90–95% have type 2 diabetes (T2D) [2, 3]. Among people with T2D, nearly half will develop diabetic kidney disease (DKD), previously termed “diabetic nephropathy” [4, 5]. DKD is the most common cause of kidney failure and end-stage kidney disease (ESKD) leading to the need for kidney replacement therapy (dialysis or transplant) in the world [6, 7]. Moreover, DKD is a leading cause of cardiovascular disease and overall mortality in people with diabetes [8, 9]. Given the ever-increasing prevalence of T2D, early detection and proper management of DKD is of great clinical importance. This review provides an update on DKD pathophysiology, clinical manifestations, and recent breakthroughs in DKD therapies.

## Pathophysiology

**Fig. 1:**
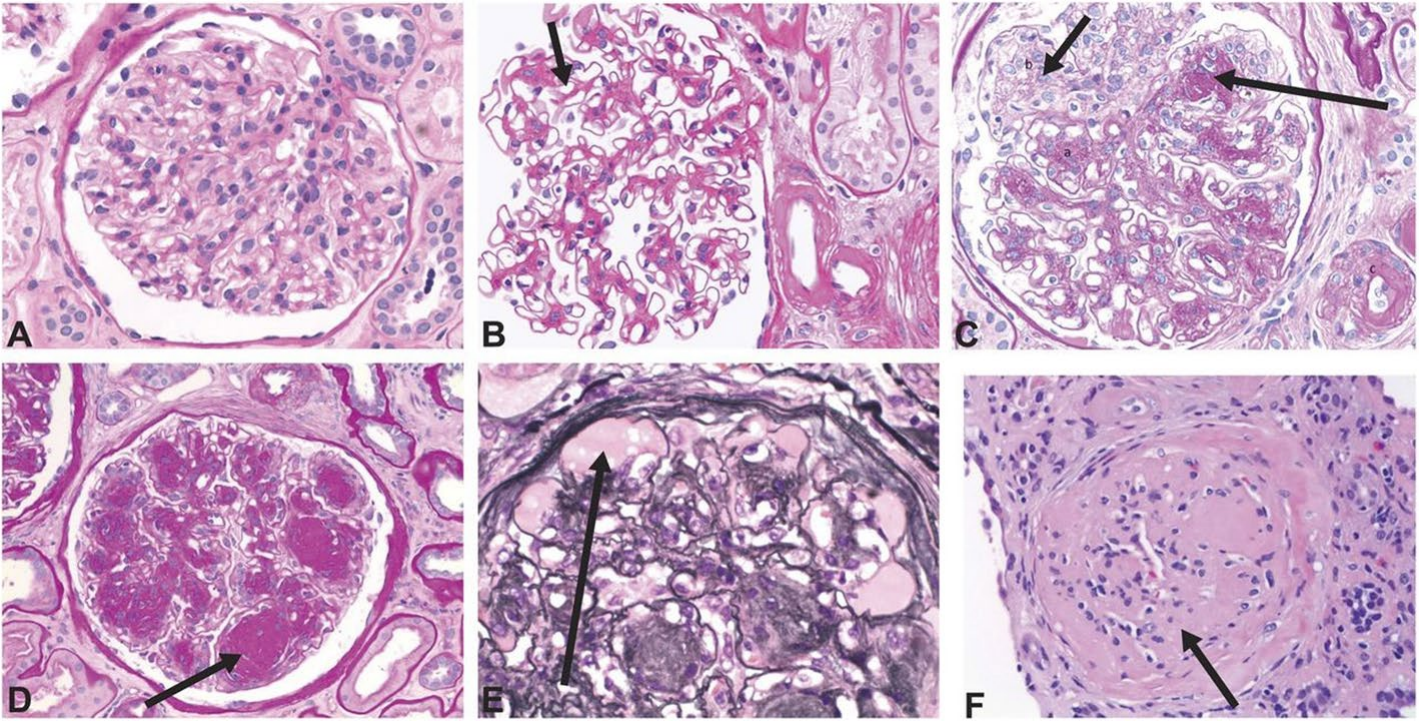
Histology images showing structural changes related to diabetic glomerulopathy. **A** Normal glomerulus. **B** Diffuse mesangial expansion with mesangial cell proliferation. **C** Prominent mesangial expansion with early nodularity and mesangiolysis. **D** Accumulation of mesangial matrix forming Kimmelstiel-Wilson nodules. **E** Dilation of capillaries forming microaneurysms, with subintimal hyaline (plasmatic insudation). **F** Obsolescent glomerulus. **A**–**D** and **F** were stained with period acid-Schiff stain. **E** was stained with Jones stain. Original magnification ×400. Reprinted with permission from American Society of Nephrology (Alicic et al., Diabetic Kidney Disease: Challenges, Progress, and Possibilities; CJASN 2017; 12; (2032-45) [11]

**Fig. 2:**
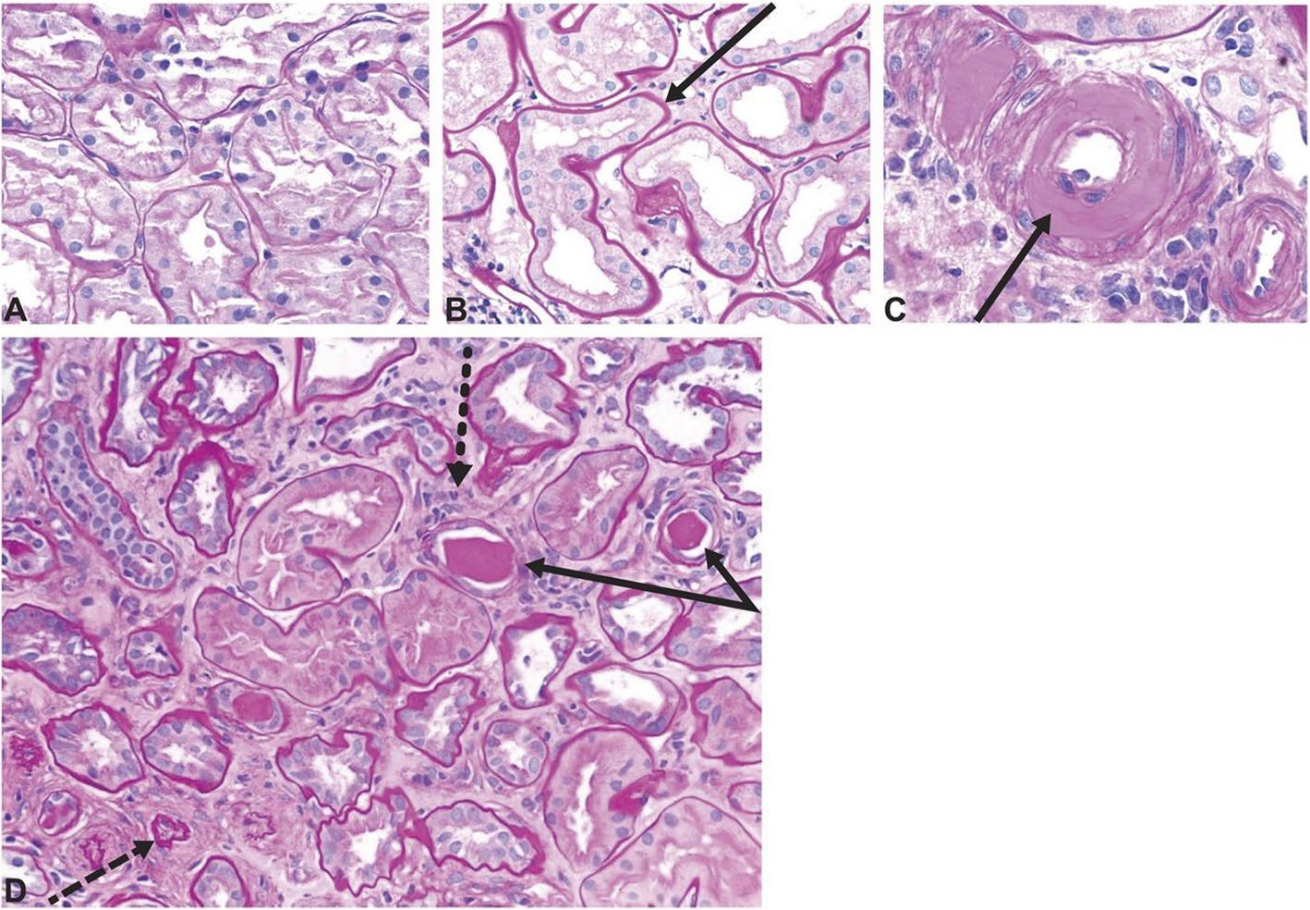
Histology images showing tubulointerstitial changes seen in diabetic kidney disease. **A** Normal kidney cortex. **B** Thickened tubular basement membrane and interstitial widening. **C** Arteriole with an intimal accumulation of hyaline material with significant luminal compromise. **D** Renal tubules and interstitium in advancing diabetic kidney disease, with thickening and wrinkled tubular basement membranes (solid arrows), atrophic tubules (dashed arrow), some containing casts, and interstitial widening with fibrosis and inflammatory cells (dotted arrow). All sections stained with period acid-Schiff stain, original magnification ×200. Reprinted with permission from American Society of Nephrology (Alicic et al. [11])

Multiple diabetes-driven pathways including hyperglycemia and associated metabolic disturbances, glomerular hemodynamic changes, and proinflammatory and profibrotic factors contribute to kidney damage in DKD [10–13]. These pathways often lead to glomerular hyperfiltration accompanied by glomerular hypertrophy, and evidence suggests that this may further lead to sclerosis, particularly with comorbid hypertension [11]. Obesity and systemic hypertension, common among people with T2D, also exacerbate glomerular hyperfiltration [14]. Arteriolar hyalinosis along with tubulointerstitial inflammation and fibrosis are also dominant features of DKD (Figs. 1 and 2) [11]. Increasing permeability to albumin, marked by high levels of albuminuria, results from progressive glomerular injury [15]. Albuminuria typically develops prior to loss of filtration, but eGFR decline may also occur without the occurrence of albuminuria in DKD [16–18]. In people who experience a decline in eGFR without albuminuria, the kidney tissue typically shows prominent vascular lesions and interstitial fibrosis [18]. Table 1 provides a description of typical findings of glomerular lesion biopsies common in DKD.

**Table 1:** Overview of classes and biopsy findings seen in glomerular lesions associated with diabetic kidney disease (DKD)

Class	Biopsy findings
I	Thickening of glomerular basement membrane >430 nm in males ages 9 years and older, >395 nm in females ages 9 years and older
II	Mild to severe expansion of mesangial extracellular material: width of interspace exceeds two mesangial cell nuclei in two or more glomerular lobules; also known as “diffuse diabetic glomerulosclerosis”
III	Nodular sclerosis, Kimmelstiel-Wilson lesions: focal, lobular, mesangial lesions with acellular, hyaline/matrix core. Generally, these lesions indicate transition from early to later stages diabetic kidney disease
IV	More than 50% global glomerulosclerosis attributed to diabetes: fibrotic lesions with a build-up of extracellular matrix proteins in the mesangial space. Presence indicates advanced diabetic kidney disease
Other changes, lesions	Interstitial fibrosis and tubular atrophy; hyalinosis of the efferent, and possibly the afferent, arterioles; insudative lesions known as “capsular drop lesions” when found in Bowman’s capsule, as “hyalinized afferent and efferent arterioles when found in the afferent and efferent arterioles, and as fibrin cap lesions or hyalinosis when found in glomerular capillaries; “tip lesion” refers to abnormality in the tubuloglomerular junction, with atrophic tubules and no visible glomerular opening, and related to advanced DKD and macroalbuminuria

Source: Tervaert et al. [19]

## Clinical manifestations

DKD often progresses to kidney failure or leads to cardiovascular events that cause death in about half of those affected [11, 20]. Therefore, early awareness, detection, and intervention are essential to improve clinical outcomes.

### Diagnostic tools and laboratory practices for DKD

A persistent elevation in urinary albumin to creatinine ratio (UACR, ≥30mg/g [≥3 mg/mmol]), and/or a persistent reduction in eGFR (<60 mL/min/1.73m^2^) in a person with diabetes indicates DKD [21]. To qualify as DKD, however, these lesions must be due only to diabetes-related factors [21].

The American Diabetes Association (ADA) Standards of Medical Care recommends that people with T2D be screened for DKD at their initial diagnosis and annually thereafter [21].

**Fig. 3:**
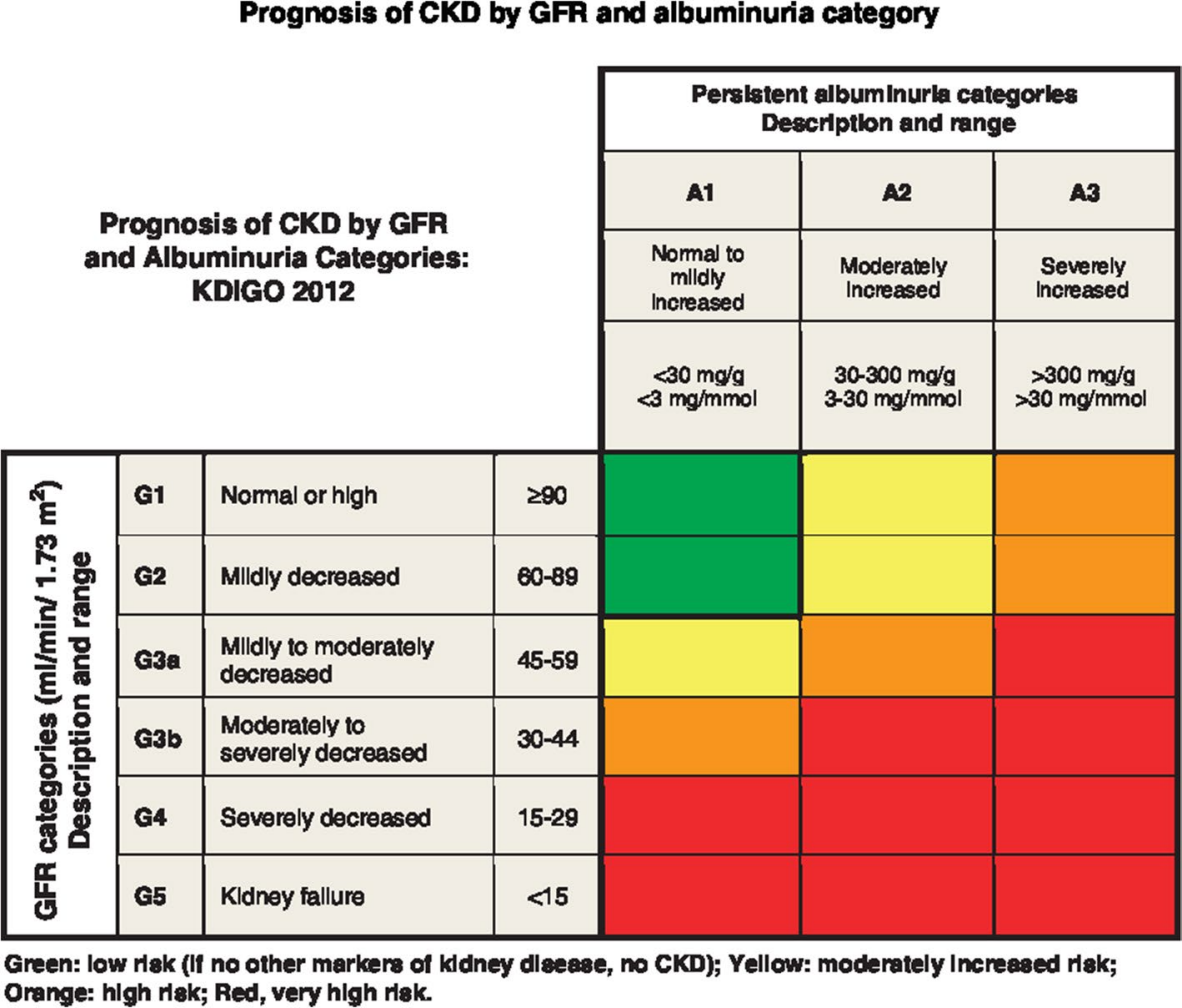
Prognosis of chronic kidney disease by GFR and albuminuria category. This figure was developed by Kidney Disease Improving Global Outcomes (KDIGO) [22] and reproduced with permission from KDIGO

As shown in Fig. 3, there are three categories of albuminuria [22]:

- *Stage A1, normal to mildly increased albuminuria*: <30 mg/g (<3 mg/mmol) UACR in urine sample
- *Stage A2, moderately increased albuminuria*, microalbuminuria: 30–300 mg/g (3–30 mg/mmol) UACR; occurring ≥2 times, 3–6 months apart [21]. This low-grade albuminuria is a less effective predictor of disease progression than macroalbuminuria [23]
- *Stage A3, severely increased albuminuria*, macroalbuminuria: >300 mg/g (>30 mg/mmol) UACR; occurring ≥2 times, 3–6 months apart [21]

The Chronic Kidney Disease Epidemiology Collaboration (CKD-EPI) equation is the most commonly used formula to estimate GFR from the serum creatinine. Recently, the American Society of Nephrology and the National Kidney Foundation have made recommendations to use race-agnostic methods excluding race in the equation to diagnose and classify chronic kidney disease as a path toward equitable healthcare [24, 25]. A major development is a new CKD-EPI 2021 eGFR equation. This new equation does not include a term for race, with the intent to increase awareness of chronic kidney disease as well as to encourage more timely detection and therapeutic interventions, for all groups of people. Addition of the serum cystatin-C to the CKD-EPI 2021 eGFR equation improves accuracy and precision [25]. Although the serum cystatin-C test is available in some regions of the world, it is not widely used yet due to costs and lack of assay standardization [26–29]. Albuminuria and decreased eGFR, in both general and high-risk populations, are also associated with increased risks for cardiovascular events and mortality, as well as all-cause mortality [30, 31].Therefore, as a holistic approach to assess kidney and cardiovascular risks, these tests should be checked at least twice a year in people with diabetes and UACR >30 mg/g (>3 mg/mmol) and/or eGFR <60 mL/min/1.73 m^2^ [21].

In addition to monitoring for kidney damage and function, people with T2D should have their glycated hemoglobin (HbA1c) tested every 3–6 months to monitor their blood glucose control [32]. The ADA recommends that people with T2D work with their physician to set an individualized goal for glycemic control avoiding hypoglycemia, but with a general target of HbA1c <7% (53 mmol/mol) [32].

## Treatments and medications

**Fig. 4:**
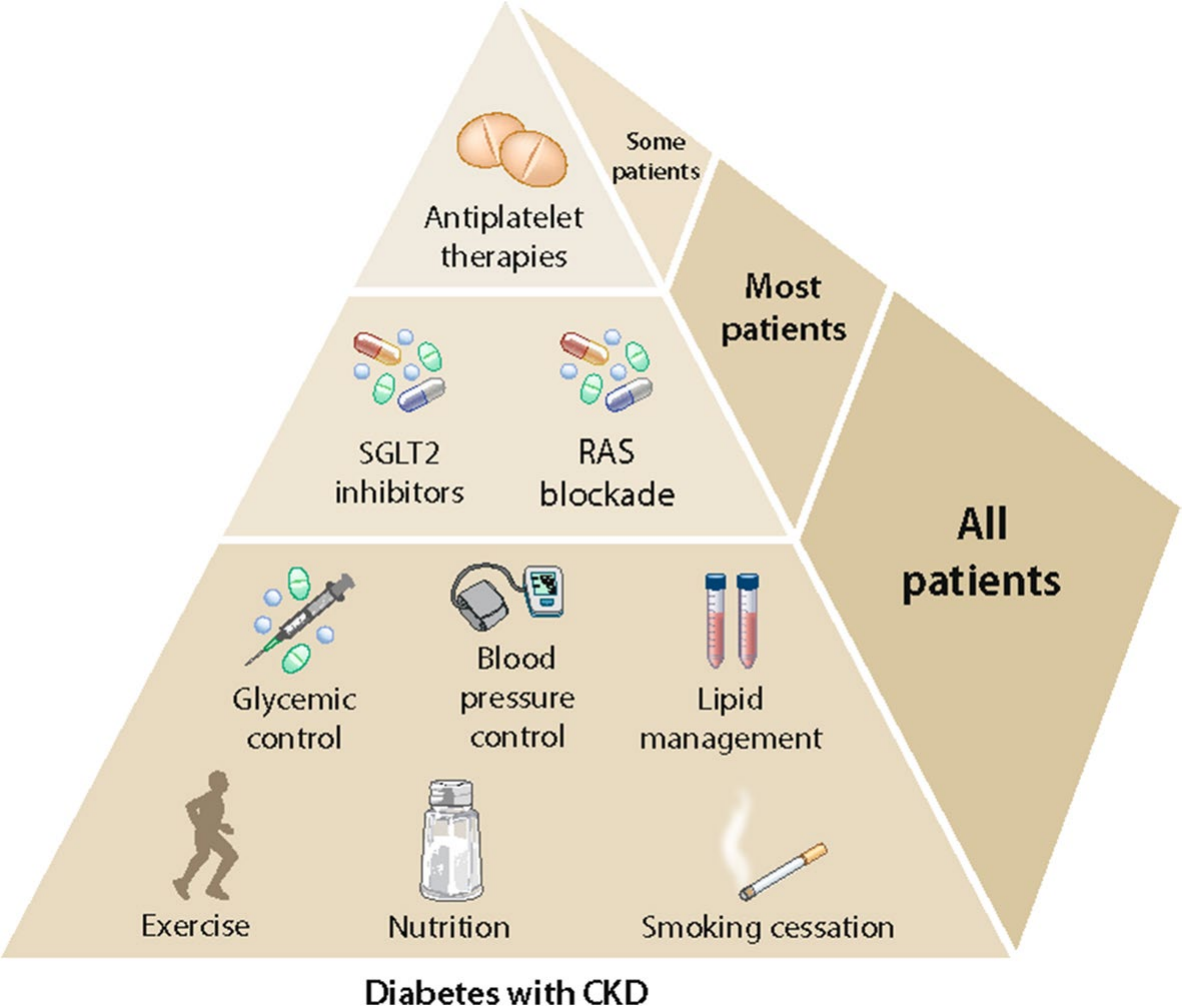
Clinical strategies to prevent development/progression of chronic kidney disease in people with diabetes. This figure was developed by Kidney Disease Improving Global Outcomes (KDIGO) [27] and reproduced with permission from KDIGO. Abbreviations: SGLT2, sodium glucose transport protein 2; RAS, renin-angiotensin system; CKD, chronic kidney disease

Several strategies exist that can help prevent DKD development and slow its progression [8, 33]. While healthy lifestyle changes are foundational, achieving optimal glycemic, blood pressure, and cholesterol levels generally require use of medications. A summary of the Kidney Disease Improving Global Outcomes (KDIGO) guideline for people with chronic kidney disease and diabetes is shown in Fig. 4.

Current goals/targets for people with T2D are:

1. Manage glycemic control—goal HbA1C ≤7% (53 mmol/mol) [32]
2. Control blood pressure—the ADA recommends blood pressure below 140/90 mmHg for people with diabetes, with a lower target (e.g., 130/80 mmHg) potentially beneficial for those with macroalbuminuria [21]. KDIGO recommends treating to a target systolic blood pressure of <120 mmHg, as tolerated, in people with chronic kidney disease with or without diabetes, but not those having had a kidney transplant or on dialysis [34]. Measures to control blood pressure should include use of either:
  i. Angiotensin-converting enzyme inhibitors (ACEi) or
  ii. Angiotensin II receptor blockers (ARB) [22]
3. Manage cholesterol levels—ideally, low density lipoprotein (LDL) of <100 mg/dL (2.59 mmol/L), total cholesterol of <150 mg/dL (3.88 mmol/L)
  i. Statins—used to treat high cholesterol [35, 36]
4. Lifestyle changes—weight reduction, increased physical activity, and smoking cessation [8, 27]

In addition to the beneficial effects that blood pressure lowering medications have on progression of DKD [37], other types of medications are also used to manage DKD in people with T2D. Table 2 lists classes, examples, and modes of action of these medications. Optimal management of blood glucose is the first step in preventing the onset of DKD. Both sodium glucose transport protein 2 inhibitors (SGLT2i) and glucagon-like peptide-1 receptor agonists (GLP-1 RA) have shown beneficial effects on DKD, such as a reduction in albuminuria or lower risk of new-onset albuminuria, largely beyond glycemic control [44, 51].

**Table 2:** Medications used in type 2 diabetes and their role in managing diabetic kidney disease

Drug class	Example(s)	Mechanism/action	Evidence of kidney protective effects	GFR range (ml/min/ 1.73m^2^)
Biguanides	Metformin	Reduces hepatic gluconeogenesis [38]	No	>30, lower dose if 30–45
Sulfonylureas	Glipizide Gliclazide Glimepiride Glyburide	Stimulates insulin secretion [39]	No	Varies by agent; generally >30
Sodium glucose transport protein-2 inhibitors (SGLT-2i)	Canagliflozin Dapagliflozin Empagliflozin Ertugliflozin	Inhibits glucose reabsorption in the kidney thereby lowering blood glucose [40]	Yes (See Table 3, discussion)	Varies by agent; generally >20
Glucagon-like Peptide Receptor Agonist (GLP-1 RA)	Exenatide Exenatide ER Liraglutide Albiglutide Dulaglutide Semaglutide	Induces insulin secretion, reduces glucagon release, lowers hepatic gluconeogenesis, slows gastric emptying [50]	Yes (See Table 4, discussion)	Varies by agent; generally >15; Exenatide is contraindicated for GFR <30 or ESKD
Insulin	Degludec Glargine Detemir NPH Aspart Lispro Glulisine Regular		No	No restriction by GFR, but doses usually must be reduced for GFR <30
Dipeptidyl peptidase-4 (DPP4) inhibitors	Sitagliptin Alogliptin Linagliptin Vildagliptin	Prevent GLP-1 degradation, thereby lowering blood glucose [61]	No	Varies by agent; generally >30 except for linagliptin which can be used with lower GFR
Thiazolidinediones	Pioglitazone	Nuclear transcription regulator and insulin sensitizer [62]	No	No restriction by GFR; watch for worsened fluid retention if eGFR <30

*Abbreviations: GFR* glomerular filtration rate, *ESKD* end-stage kidney disease, *eGFR* estimated glomerular filtration rate

Tables 3, 4, and 5 provide summaries of recent clinical trials of agents (SGLT-2i, GLP-1 RA, and non-steroidal mineralocorticoid receptor antagonists, MRAs) showing promise in managing DKD.

**Table 3:** Recent clinical trials of SGLT-2i agents with kidney outcomes

Study	Inclusion criteria	Participants	Kidney outcome(s)	HR (95% CI) or other as specified
**Studies with at least one primary kidney outcome**				
CREDENCE [41] Feb 2014–Oct 2018 695 sites in 34 countries [42]	Adults with T2D, HbA1c 6.5% to 12.0%, age ≥30 yrs, eGFR (CKD-EPI) 30 to ≤90 AND UACR 300-5000, taking stable dose of ACEi or ARB for ≥4 weeks prior to randomization	*N*=2202 100 mg canagliflozin once daily *N*=2199 placebo once daily BL: mean age 63 yrs, 66% male, 67% white, mean HbA1c 8.3%, mean duration T2D 16 yrs, mean eGFR 56, median UACR 927	A) Primary kidney composite outcome of ESKD (dialysis for ≥30 days or kidney transplantation or eGFR≤15), doubling of serum creatinine from BL sustained for ≥30 days, or death from kidney or CVD cause B) Secondary kidney composite outcome of ESKD, doubling of serum creatinine, or kidney death C) ESKD D) Doubling of serum creatinine E) Dialysis or kidney transplantation F) Kidney death G) ESKD, kidney- or CVD-related death Dialysis, kidney transplantation, or kidney death	A) 0.70 (0.59–0.82) B) 0.66 (0.53–0.81) C) 0.68 (0.54–0.86) D) 0.60 (0.48–0.76) E) 0.74 (0.55–1.00) F) – G) 0.73 (0.61–0.87) 0.72 (0.54–0.97)
DAPA-CKD [43] Feb 2017–June 2020 386 sites in 21 countries	Adults with or without T2D, an eGFR of 25-75 AND a UACR of 200–5000, taking stable dose of ACEi or ARB >4 weeks prior to screening	*N*=2152 10mg dapagliflozin once daily *N*=2152 placebo once daily BL: mean age 62 yrs, 67% male, 53% white, 68% T2D, mean eGFR 43, 48% had UACR >1000	A) Primary kidney composite outcome of decline of at least 50% in eGFR or death from kidney or CV cause in participants **overall**; B) Primary kidney composite outcome of decline of at least 50% in eGFR or death from kidney or CV cause in participants **with** T2D; C) Primary kidney composite outcome of decline of at least 50% in eGFR or death from kidney or CV cause in participants **without** T2D D) Secondary kidney outcomes: composite of sustained eGFR decline of at least 50%, ESKD, kidney death; Between-group difference in LS mean slope of eGFR from BL to month 30	A) 0.61 (0.51–0.72) B) 0.64 (0.52–0.79) C) 0.50 (0.35–0.72) D) 0.56 (0.45–0.68) Difference = 0.93 mL/min/ 1.73m^2^/yr (0.61–1.25)
**Studies with kidney outcomes as secondary outcome(s) only**				
EMPA-REG OUTCOME [44] July 2010–April 2015 590 sites in 42 countries	Adults with T2D, HbA1c 7.0 to 10% if on antidiabetic therapy or 7 to 9% for drug naïve, age ≥18 yrs, established CVD or high risk for CVD, eGFR (MDRD) ≥30	*N*=4685 empagliflozin (10 or 25 mg) once daily *N*=2333 placebo once daily BL: mean age 64.5 yrs, 70% male, 72% white, mean HbA1c 8.1% [45]	A) Incident or worsening nephropathy (UACR >300) B) Doubling of serum creatinine AND eGFR ≤45 C) Initiation of kidney replacement D) Composite outcome of incident or worsening nephropathy or CV-related death E) Progression to macroalbuminuria F) Composite of b + c + kidney-related death G) Incident albuminuria (UACR≥30) in those with normal albuminuria at BL	A) 0.61 (0.53–0.70) B) 0.56 (0.39–0.79) C) 0.45 (0.21–0.97) D) 0.61 (0.55–0.69) E) 0.62 (0.54–0.72) F) 0.54 (0.40–0.75) G) 0.95 (0.87–1.04)
CANVAS, CANVAS-R [46] Dec 2009–Feb 2017 667 sites in 30 countries	Adults with T2D, HbA1c 7% to 10.5%, eGFR ≥30, age ≥30 yrs with symptomatic history of CVD, or age ≥50 yrs with 2+ risk factors for CVD	*N*=5795 canagliflozin (100 or 300 mg) *N*=4347 placebo BL: mean age 63.3 yrs, 64% male, 78% white, mean duration T2D=14 yrs, mean HbA1c 8.2%	A) Composite of progression of albuminuria (more than 30% increase in albuminuria), change from either normoalbuminuria to microalbuminuria or micro- to macroalbuminuria B) Regression of albuminuria C) Composite of 40% reduction in eGFR for at least 2 consecutive measures, need for kidney replacement therapy, and kidney-related death	A) 0.73 (0.67–0.79) B) 1.70 (1.51–1.91) C) 0.60 (0.47–0.77)
DECLARE-TIMI 58 [47] April 2013–Sept 2018 882 sites, 33 countries	Adults with T2D, HbA1c 6.5% to 11.9%, age ≥40 yrs, creatinine clearance ≥60 ml/min, with multiple CVD risk factors or established CVD	*N*=8582 dapagliflozin (10 mg once daily) *N*=8578 placebo (once daily) BL: mean age 64 yrs, 63% male, 80% white, mean HbA1c 8.3%, median duration T2D 10.5 yrs, mean eGFR 85	A) Composite of sustained decrease in eGFR (per CKD-EPI) of 40% or more to less than 60, new ESKD, or death from kidney or CV cause B) Sustained decrease in eGFR (per CKD-EPI) of 40% or more to less than 60, new ESKD, or death from kidney cause	A) 0.76 (0.67–0.87) B) 0.53 (0.43–0.66)
VERTIS-CV [48] Nov 2013–Dec 2019 567 sites in 34 countries	Adults, with T2D and established atherosclerotic CVD, age ≥40 yrs, HbA1c 7.0% to 10.5%, BMI≥18 kg/m^2^, eGFR ≥30	*N*=5499 5 or 15 mg ertugliflozin once daily *N*=2747 placebo once daily BL: mean age 64 yrs, 70% male, 88% white, mean HbA1c 8.2%, mean duration T2D 13 yrs, mean eGFR 76	Composite of kidney death, kidney replacement therapy, or doubling of serum creatinine	0.81 (0.63–1.04)
EMPEROR REDUCED [49] March 2017–May 2020 520 sites in 20 countries	Adults with chronic heart failure and left ventricular ejection fraction <40%, age ≥18 yrs Note: Roughly 7 in 10 participants were taking MRAs at BL	*N*=1863 10 mg empagliflozin once daily *N*=1867 placebo once daily BL: mean age 67 yrs, 76% male, 70% white, 50% DM, mean eGFR 62	A) Rate of decline in eGFR calculated per CKD-EPI equation B) Composite kidney outcome of chronic dialysis or kidney transplantation, profound & sustained reduction in eGFR	A) Between group difference=1.73 ml/min/1.73m^2^ (1.10–2.37) B) 0.50 (0.32–0.77)

*Abbreviations: SGLT-2i*, sodium glucose transport protein 2 inhibitor; *HR*, hazard ratio; *CI*, confidence interval; *EMPA-REG OUTCOME*, Empagliflozin Cardiovascular Outcome Event Trial in Type 2 Diabetes Mellitus Patients, *T2D* type 2 diabetes, *HbA1c* glycated hemoglobin, *CVD* cardiovascular disease, *eGFR* estimated glomerular filtration rate, in mL/min/1.73 m^2^ body surface area, *MDRD* Modification of Diet in Renal Disease, *BL* baseline, *yrs* years, *UACR* urine albumin to creatinine ratio, in mg albumin to g creatinine, *CV* cardiovascular, *CANVAS*, *CANVAS-R* Canagliflozin Cardiovascular Assessment Study, *DECLARE-TIMI 58* Dapagliflozin Effect on Cardiovascular Events-Thrombolysis in Myocardial Infarction 58, *CKD-EPI* chronic kidney disease epidemiology collaboration, *ESKD* end-stage kidney disease, *VERTIS-CV* EValuation of ERTugliflozin effIcacy and safety – CardioVascular outcomes, *BMI* body mass index, *EMPEROR REDUCED* Empagliflozin Outcome Trial in Patients with Chronic Heart Failure, Reduced Ejection Fraction. *MRA* mineralocorticoid receptor antagonist, *DM* diabetes mellitus, *CREDENCE* Canagliflozin and Renal Events in Diabetes with Established Nephropathy Clinical Evaluation, *ACEi* angiotensin-converting enzyme inhibitor(s), *ARB* angiotensin II receptor blocker(s), *DAPA-CKD* Dapagliflozin and Prevention of Adverse Outcomes in Chronic Kidney Disease, *LS* least-square, *BL* baseline

**Table 4:** Recent clinical trials of GLP-1 RA agents with kidney outcomes

Study	Inclusion criteria	Participants	Kidney outcome	HR (95% CI) or other as specified
**Studies with kidney outcomes as secondary outcome(s) only**				
LEADER [51] Aug 2010–Dec 2015 410 sites in 32 countries	Adults with T2D, age ≥50 yrs with established CVD, or age ≥60 yrs with CVD risk factors, HbA1c ≥7%, no GLP-1 RA or DPP-4i for 3 months prior to screening	*N*=4668 maximum 1.8mg liraglutide (as tolerated) once daily *N*=4672 placebo once daily BL: mean age 64 yrs, 64% male, 78% white, mean HbA1c 8.7%, mean duration T2D 13 yrs, mean eGFR (MDRD) 80, 26% had microalbuminuria, 11% had BL macroalbuminuria	A) Composite of new onset persistent macroalbuminuria, persistent doubling of serum creatine, kidney replacement therapy, death from kidney causes B) New onset persistent macroalbuminuria C) Persistent doubling of serum creatinine D) Kidney replacement therapy E) Death from kidney cause F) Decline in eGFR over 36 months G) Increase in UACR H) New onset microalbuminuria	A) 0.78 (0.67–0.92) B) 0.74 (0.60–0.91) C) 0.90 (0.67–1.20) D) 0.87 (0.61–1.25) E) 1.60 (0.52–4.90) F) Between group difference=1.02 (*p*=0.01) G) Between group difference=0.83 (*p*<0.001) H) 0.87 (0.83–0.93)
REWIND [52] July 2011–Aug 2018 371 sites in 24 countries	Adults with T2D, age ≥50 with previous CVD event or with CVD risk factors, HbA1c ≤9.5%	*N*=4949 1.5mg dulaglutide once weekly *N*=4952 placebo once weekly BL: mean age 66 yrs, 54% male, 76% white, mean duration T2D 11 yrs, mean HbA1c 7.4%, mean eGFR 77	A) Composite of development of macroalbuminuria (UACR>33.9 mg/mmol), sustained decline in eGFR ≥30%, or new chronic kidney replacement therapy B) Development of macroalbuminuria C) Sustained decline in eGFR ≥30% D) New chronic kidney replacement therapy	A) 0.85 (0.77–0.93) B) 0.77 (0.68–0.87) C) 0.89 (0.78–1.01) D) 0.75 (0.39–1.44)
Harmony Outcomes [53] July 2015–March 2018 610 sites in 28 countries	Adults with T2D and established CVD, age ≥40 yrs, HbA1c >7%, eGFR (MDRD) ≥30, not using GLP-1 RA at screening	*N*=4731 30–50 mg albiglutide as tolerated once weekly *N*=4732 placebo once weekly BL: mean age 64 yrs, 70% male, 70% white, mean duration T2D 14 yrs, mean HbA1c 8.7%, mean eGFR 79	Change in eGFR by treatment group	Mean eGFR difference=−1.11 (−1.84 to −0.39) at 8 months and -0.43 (−1.26 to 0.41) at 16 months. Figure 4 shows significant difference (no CI overlap) favoring albiglutide at 28 months but no numbers provided
SUSTAIN-6 [54] Feb 2013–March 2016 230 sites in 20 countries	Adults with T2D, age ≥50 yrs with established CVD, heart failure (NYHA class II or III), or chronic kidney failure OR age ≥60 yrs with one or more CVD risk factors, HbA1c ≥7%, no use of DPP-4i within 30 days prior to screening or GLP-1 RA within 90 days prior to randomization	*N*=1648 0.5mg or 1.0 mg semaglutide once weekly *N*=1649 placebo once weekly BL: mean age 65 yrs, 61% male, 83% white, 30% eGFR>90, mean HbA1c 8.7%, mean T2D duration 14 yrs	A) New or worsening nephropathy B) Persistent macroalbuminuria C) Persistent doubling of serum creatinine and creatinine clearance per MDRD <45 D) Need for continuous kidney replacement therapy	A) 0.64 (0.46–-0.88) B) 0.54 (0.37–0.77) C) 1.28 (0.64–2.58) D) 0.91 (0.40–2.07)
AMPLITUDE-O [55] April 2018–Dec 2020 344 sites in 28 countries	Adults with T2D, HbA1c >7%, age ≥18 yrs with history of CVD, **OR** males ≥50 yrs/females ≥55 yrs with eGFR (MDRD) 25.0 to 59.9 and ≥1 CV risk factor, no use of GLP-1 RA or DPP-4i within 3 months prior to screening	*N*=1359 initial dose 2mg efpeglenatide once weekly, titrated to 4mg or 6mg once daily to study end *N*=1358 6mg efpeglenatide once weekly *N*=1359 placebo once weekly BL: mean age 65 yrs, 67% male, 87% white, mean HbA1c 8.9%, mean eGFR 72, mean duration T2D 15 yrs, median UACR 28.3	A) Incident macroalbuminuria B) Between-group difference in UACR C) LS mean difference in eGFR D) Decrease in eGFR≥40% for ≥30 days, ESKD, or all-cause death E) Composite of MACE, death from non-CV cause, hospitalization for heart failure, or occurrence of (A)	A) 0.68 (0.57–0.79) B) 0.68 (0.58–0.80) C) Lower by 21% (14–28%) D) Higher by 0.9 (0.3–1.50) E) 0.77 (0.57–1.02) F) 0.71 (0.59–0.87)
ELIXA [56] June 2010–Feb 2015 829 sites in 49 countries [57]	Adults with T2D, HbA1c 5.5% to 11.0%, age≥30 yrs, with acute coronary syndrome (STEMI, non-STEMI, or unstable angina) <180 days before screening, HbA1c 5.5 to 11%, and eGFR (MDRD) ≥30, taking GLP-1 RA or DPP-4i during study	*N*=3034 10μg lixisenatide increased up to 20μg once daily *N*=3034 placebo once daily BL: mean age 60 yrs, 69% male, 75% white, mean duration T2D 9 yrs, mean HbA1c 7.7%, mean eGFR 76	Percent change in UACR from BL to study week 108 (BL UACR and study week 108 data available for *n*=2830 placebo, *n*=2803 lixisenatide)	+34% placebo, +24% lixisenatide, *p*<0.01, adjusted for BL UACR, treatment, region, BL use of ACEi and ARB; +32% placebo, +26 lixisenatide, *p*=0.07, adjusted for BL and 3-month HbA1c
EXSCEL [58] June 2010–April 2017 688 sites in 35 countries [59]	Adults with T2D, HbA1c 6.5% to 10.0%, age≥18 yrs, eGFR (MDRD) ≥30, range of CV risk factors, taking 0 to 3 oral glycemic control drugs or insulin with or without use of 1–2 oral glycemic drugs, never used GLP-1 RA	Propensity score matched *N*=572 placebo; *N*=572 exenatide once weekly +SGLT2i; *N*=575 exenatide once weekly; N=575 exenatide once weekly + SGLT2i BL: mean age 63 yrs, 62% male, 76% white, mean duration T2D 12 yrs, mean HbA1c 8%	Outcome comparisons between 1: placebo only with exenatide + SGLT2i, and 2: exenatide only with exenatide + SGLT2i: A) Change over time in eGFR (per MDRD) B) Composite of persistent 40% reduction in eGFR, kidney dialysis, or kidney transplant C) Composite of “B” plus new macroalbuminuria	A) (1) 1.94 (0.94–2.94); (2) 2.38 (1.40–3.35) B) (1) 0.32 (0.06–1.59); (2) 0.21 (0.05–0.97) C) (1) 0.43 (0.15–1.22); (2) 0.35 (0.13–0.98)
AWARD 7 [60] July 2012–Dec 2016 99 sites in 9 countries	Adults with T2D and stage 3 or 4 CKD, age≥18 yrs, HbA1c 7.5% to 10.5%, taking insulin alone or with oral glucose control drug, taking maximum tolerated dose of ACEi or ARB, not taking GLP-1 RA or DPP-4i	*N*=192 1.5mg dulaglutide once weekly; *N*=190 0.75mg dulaglutide once weekly; *N*=194 insulin glargine once daily BL: mean age 65 yrs, 69% white, 52% male, mean HbA1c 8.6%, mean duration T2D 18 yrs, mean eGFR (CKD-EPI) by creatinine 36 (35 by cystatin C), median UACR = 214 for dulaglutide 1.5mg, = 234 for dulaglutide 0.75mg, = 196 for insulin glargine	Outcome comparisons between (1) insulin glargine vs dulaglutide 1.5mg, and (2) insulin glargine vs dulaglutide 0.75mg A) Change in eGFR per CKD-EPI creatinine B) Change in eGFR per CKD-EPI cystatin C C) UACR change from BL D) Change in eGFR per MDRD E) Kidney events of increase in serum creatinine >30% from BL, ESKD	A) Week 26 LS mean change (1): −0.1 (*p*<0.05), (2): −0.4 (*p*<0.05) Week 52 Change (1): −1.1 (ns), (2): −1.5 (ns) B) Week 26 LS mean change (1): 0.8 (*p*<0.05), (2): 1.1 (*p*<0.0001); Week 52 Change (1): −0.7 (*p*<0.05), (2): −0.7 (*p*<0.05) C) Week 26 Among those with BL macro-albuminuria, UACR decreased for dulaglutide 1.5mg vs insulin by 43.1% (*p*=0.008) at week 26 and decreased by 29% (*p*=0.02) at week 52; for dulaglutide 0.75mg, a decrease of 25.3% (no p-value provided) at week 26 and decrease of 12.3 (no p-value provided) at week 52; for those without BL macroalbuminuria, decrease of 0.4% at week 26 (ns) and decrease of 3.4 % (ns) at week 52 for dulaglutide 1.5mg; for dulaglutide 0.75mg, decrease of 18% (ns) at week 26 and decrease of 15.3% (ns) at week 52 D) Week 26 LS mean change (1): no change (*p*<0.05), (2): −0.2 (*p*<0.05); Week 52 change (1): −0.4 (*p*<0.05), (2) −1.3 (ns) E) Number of events Dulaglutide 1.5mg = 79 (41%); Dulaglutide 0.75mg = 73 (38%); Insulin = 91 (47%)

Abbreviations: *GLP-1 RA* glucagon-like peptide-1 receptor agonist, *HR* hazard ratio, *CI* confidence interval, *LEADER* Liraglutide Effect and Action in Diabetes: Evaluation of Cardiovascular Outcome Results, *T2D* type 2 diabetes, *CVD* cardiovascular disease, *HbA1c* glycated hemoglobin, *DPP-4i* dipeptidyl peptidase-4 inhibitor, *BL* baseline, *yrs* years, *eGFR* estimated glomerular filtration rate, in mL/min/1.73 m^2^ body surface area, *MDRD* Modification of Diet in Renal Disease, *UACR* urine albumin to creatinine ratio, in mg albumin to g creatinine, *Harmony Outcomes* Effect of albiglutide, when added to standard blood glucose lowering therapies, on major cardiovascular events in subjects with type 2 diabetes, *REWIND* Dulaglutide and Cardiovascular Outcomes in Type 2 Diabetes, *SUSTAIN-6* Trial to Evaluate Cardiovascular and Other Long-term Outcomes with Semaglutide in Subjects with Type 2 Diabetes, *NYHA* New York Heart Association, *AMPLITUDE-O* Effect of Efpeglenatide on Cardiovascular Outcomes, *CV* cardiovascular, *MACE* major adverse cardiovascular events, *ELIXA* Evaluation of Lixisenatide in Acute Coronary Syndrome, *STEMI* ST-elevation myocardial infarction, *ACEi* angiotensin-converting enzyme inhibitor, *ARB* angiotensin II receptor blocker, *EXSCEL* Exenatide Study of Cardiovascular Event Lowering, *SGLT2i* sodium glucose transport protein 2 inhibitor, *AWARD 7* Assessment of Weekly Administration of LY2189265 (dulaglutide) in Diabetes, *CKD-EPI* chronic kidney disease epidemiology collaboration, *ESKD* end-stage kidney disease, *LS* least squares, *ns* non-significant, *BL* baseline

**Table 5:** Recent clinical trials of MRA agents with kidney outcomes

Study	Inclusion criteria	Participants	Kidney outcome	HR (95% CI) or other as specified
**Studies with at least one primary kidney outcome**				
FIDELIO DKD [63] Sept 2015–April 2020 978 sites in 48 countries	Adults with T2D and CKD (UACR 30 to <300 AND eGFR (CKD-EPI) 25 to <60 **OR** UACR 300-5000 AND eGFR 25 to <75), age ≥18 yrs, taking maximum tolerated dose of ACEi or ARB, serum potassium ≤4.8 mmol/L, HbA1c ≤12%	*N*=2833 finerenone, 10mg once daily titrated up to 20mg once daily as tolerated *N*=2841 placebo once daily BL: mean age 66 yrs, 70% male, 63% white, mean duration T2D 17 yrs, mean HbA1c 7.7%, mean eGFR 44, median UACR 852, mean serum potassium 4.37 mmol/L, 7% taking GLP-1 RA, 5% taking SGLT2i	Primary outcomes: A) Kidney composite of kidney failure (ESKD or eGFR <15), sustained decrease of ≥40% in eGFR from BL for ≥4 weeks, or kidney-related death B) Kidney failure C) ESKD D) eGFR <15 E) Sustained decrease of ≥40% in eGFR from BL for ≥4 weeks F) Kidney-related death Secondary outcomes G) Change in UACR from BL to study month 4 H) Composite of kidney failure, sustained decrease of ≥57% from BL eGFR for ≥4 weeks, or kidney-related death I) Sustained decrease of ≥57% from BL eGFR for ≥4 weeks	A) 0.82 (0.73–0.93) B) 0.87 (0.72–1.05) C) 0.86 (0.67–1.10) D) 0.82 (0.67–1.01) E) 0.81 (0.72–0.92) F) -- G) Between group difference=0.69 (0.66, 0.71) H) 0.76 (0.65, 0.90) A) I) 0.68 (0.55–0.82)
**Studies with kidney outcomes as secondary outcome(s) only**				
FIGARO DKD [64] Sept 2015–Feb 2021; **NOTE**: COVID-19 caused trial disruption for 29% of pts, and temporary interruption of trial regiment for 10% of pts 975 sites in 48 countries	Adults with T2D, age ≥18 yrs, HbA1c <12%, with either UACR 30 to <300 AND eGFR (per CKD-EPI) 25 to 90 **OR** UACR 300-5000 AND eGFR ≥60, taking ACEi or ARB at maximum tolerated dose, serum potassium ≤4.8 mmol/L at screening	*N*=3686 finerenone, 10mg once daily titrated up to 20mg per day as tolerated *N*=3666 placebo once daily BL: mean age 64 yrs, 69% male, 72% white, mean HbA1c 7.7%, mean eGFR 68, median UACR 308, 8% taking SGLT2i, and 8% taking GLP-1 RA at BL, with additional 16% and 11%, respectively, starting over study period	A) Composite of 1st occurrence of kidney failure (ESKD or sustained decrease in eGFR <15), sustained decrease of ≥40% from BL eGFR for ≥4 weeks, or kidney-related death B) 1st occurrence of kidney failure C) ESKD D) Sustained decrease in eGFR <15 E) sustained decrease of ≥40% from BL eGFR for ≥4 weeks F) kidney-related death G) Change in UACR from BL to study week 4 H) Composite of 1st occurrence of kidney failure, sustained decrease of ≥57% from BL eGFR for ≥4 weeks, or kidney-related death I) sustained decrease of ≥57% from BL eGFR for ≥4 weeks	B) 0.87 (0.76–1.01) C) 0.72 (0.49–1.05) D) 0.64 (0.41–0.995) E) 0.71 (0.43–1.16) F) 0.87 (0.75–1.00) G) – H) Between group difference=0.68 (0.65–0.70) I) 0.77 (0.60–0.99) J) 0.76 (0.58–1.00)

Abbreviations: *MRA* mineralocorticoid receptor antagonist, *HR* hazard ratio, *CI* confidence interval, *FIGARO DKD* FInerenone in reducinG cArdiovascular moRtality and mOrbidity in Diabetic Kidney Disease, *T2D* type 2 diabetes, *yrs* years, *HbA1c* glycated hemoglobin, *UACR* urine albumin to creatinine ratio, in mg albumin to g creatinine, *eGFR* estimated glomerular filtration rate, in mL/min/1.73 m^2^ body surface area, *CKD-EPI* chronic kidney disease epidemiology collaboration, *ACEi* angiotensin-converting enzyme inhibitor, *ARB* angiotensin II receptor blocker, *BL* baseline, *SGLT2i* sodium glucose transport protein 2 inhibitor, *ESKD* end-stage kidney disease, *FIDELIO DKD* FInerenone in reducing kiDnEy faiLure and dIsease prOgression in Diabetic Kidney Disease

### SGLT-2i agents (Table 3)

Two double-blind, randomized, placebo-control trials, Canagliflozin and Renal Events in Diabetes with Established Nephropathy Clinical Evaluation (CREDENCE) [41] and Dapagliflozin and Prevention of Adverse Outcomes in Chronic Kidney Disease (DAPA-CKD) [43], included kidney disease endpoints as the primary outcome. In CREDENCE, participants assigned to canagliflozin had a 30% reduced risk (hazard ratio (HR)=0.70 [95% confidence interval (CI): 0.59–0.82]) of the primary kidney composite outcome (ESKD, doubling of serum creatinine from baseline sustained for at least 30 days, or death from kidney or cardiovascular disease causes) as compared with participants assigned to placebo [41]. A similar effect was seen in DAPA-CKD, with participants assigned to dapagliflozin having a 39% reduced risk (HR=0.61 [95% CI: 0.51–0.72]) of the primary kidney composite outcome (>50% decline in eGFR from baseline or kidney- or CV-related death) as compared to those in the placebo arm [43]. The majority of participants in both trials were already receiving ACEi or ARBs in maximum tolerated doses where possible. Approximately one third (*n*=1398) of the participants in DAPA-CKD did not have T2D [43].

Other clinical trials with SGLT-2i investigated kidney disease outcomes as a secondary outcome. Four trials, Empagliflozin Cardiovascular Outcome Event Trial in Type 2 Diabetes Mellitus Patients (EMPA-REG OUTCOME) [44], Canagliflozin Cardiovascular Assessment Study (CANVAS, CANVAS-R) [46], Dapagliflozin Effect on Cardiovascular Events-Thrombolysis in Myocardial Infarction 58 (DECLARE-TIMI 58) [47], and Empagliflozin Outcome Trial in Patients with Chronic Heart Failure, Reduced Ejection Fraction (EMPEROR REDUCED) [49], reported lower rates of kidney disease composite outcomes in those assigned to the active drug than to placebo (EMPA-REG OUTCOME HR=0.61 [95% CI: 0.53–0.70]; CANVAS, CANVAS-R HR=0.73 [95% CI: 0.67–0.79]; DECLARE-TIMI 58 HR=0.76 [95% CI: 0.67–0.87]; EMPEROR REDUCED HR=0.50 [95% CI: 0.32–0.77]) [44, 46, 47, 49]. Composite kidney disease outcomes were somewhat similar between studies (e.g., composite of sustained decrease in eGFR of 40% or more, to less than 60 mL/min/1.73 m^2^, incident ESKD, death from kidney or cardiovascular disease causes in DECLARE-TIMI 58 and incident chronic dialysis or kidney transplantation, profound and sustained reduction in eGFR in EMPEROR REDUCED) [47, 49]. One study, eValuation of ERTugliflozin effIcacy and safety – CardioVascular outcomes (VERTIS-CV) [48], reported no significant difference in their secondary kidney disease outcome (death due to kidney disease, kidney replacement therapy, or doubling of serum creatinine) between those randomized to ertugliflozin versus placebo (HR=0.80 [95% CI: 0.61–1.05] )[48].

### GLP-1 RA agents (Table 4)

Cardiovascular outcome trials have also examined GLP-1 RA in people with T2D with kidney disease outcomes as secondary outcomes; to date, there are no published studies of GLP-1 RAs with kidney outcomes as a primary outcome. Randomized, placebo-controlled trials including Liraglutide Effect and Action in Diabetes: Evaluation of Cardiovascular Outcome Results (LEADER) [51]; Dulaglutide and Cardiovascular Outcomes in Type 2 Diabetes (REWIND) [42]; Effect of albiglutide, when added to standard blood glucose lowering therapies, on major cardiovascular events in subjects with type 2 diabetes (Harmony Outcomes) [52]; Trial to Evaluate Cardiovascular and Other Long-term Outcomes with Semaglutide in Subjects with Type 2 Diabetes (SUSTAIN-6) [53]; Exenatide Study of Cardiovascular Event Lowering (EXSCEL) [54]; Evaluation of Lixisenatide in Acute Coronary Syndrome (ELIXA) [58]; Assessment of Weekly Administration of dulaglutide in Diabetes (AWARD 7) [56]; and Effect of Efpeglenatide on Cardiovascular Outcomes (AMPLITUDE-O) [60] all reported significantly lower rates of kidney disease outcomes in participants assigned to the active drug as compared with those assigned to placebo, or active drug as compared to insulin in AWARD-7. LEADER, REWIND, and AMPLITUDE-O report significantly lower risk of composite kidney disease outcomes among those assigned to study drug versus placebo (LEADER HR=0.78 [95% CI: 0.67–0.92]; REWIND HR=0.85 [95% CI: 0.77–0.93]; AMPLITUDE-O HR=0.68 [95% CI: 0.57–0.79]) [42, 51, 60]. EXSCEL found no significant difference in risk of their composite outcome (HR=0.43 [95% CI: 0.15–1.22]) [54].

Other GLP-1RA studies reported on individual kidney disease measures. In Harmony Outcomes, there was a between-group difference (albiglutide vs. placebo) in change in eGFR at 8 months (mean difference=−1.11 [95% CI: −1.84 to 0.39]) and at 16 months (mean difference=−0.43 [95% CI: −1.26 to 0.41]) [52]. SUSTAIN-6 reported significantly lower risk of new or worsening nephropathy (HR=0.64 [95% CI: 0.46–0.88]) or persistent macroalbuminuria (HR=0.54 [95% CI: 0.37–0.77]) among those assigned to semaglutide as compared with placebo [53]. In ELIXA, participants assigned to lixisenatide had a 24% increase in UACR from baseline to study week 108 while those assigned to placebo had a 34% increase, a significant difference (*p*=0.004) [58]. In AWARD 7, participants assigned to dulaglutide had higher eGFR at 52 weeks than those assigned to insulin glargine (eGFR least square means = 34.0 mL/min/1.73m^2^, *p*=0.005 for dulaglutide 1.5 mg, eGFR least square means = 33.8 mL/min/1.73 m^2^, *p*=0.009 for dulaglutide 0.75mg) [56]. More details of these studies are provided in Table 4. As the kidney outcomes mentioned here were all secondary outcomes from cardiovascular outcomes or glycemic lowering trials, there is a clear need for studies with primary kidney disease outcomes in participants with T2D and DKD [55]. The Effect of Semaglutide Versus Placebo on the Progression of Renal Impairment in Subjects With Type 2 Diabetes and Chronic Kidney Disease (FLOW, NCT03819153) trial is investigating a GLP-1RA with a primary kidney disease outcome (≥50% eGFR decline, kidney failure, and death from kidney or CV disease) [65]. A companion study, Renal Mode of Action of Semaglutide in Patients With Type 2 Diabetes and Chronic Kidney Disease (REMODEL, NCT04865770), is examining the effect of semaglutide on kidney inflammation, perfusion, and oxygenation [66].

### MRA agents (Table 5)

Two recent clinical trials report on the effects of a non-steroidal MRA, finerenone, on kidney disease outcomes. Finerenone demonstrated positive results in FInerenone in reducing kiDnEy faiLure and dIsease prOgression in Diabetic Kidney Disease (FIDELIO-DKD) with kidney disease endpoints as primary outcomes [67]. In this study, participants assigned to finerenone had an 18% lower risk of the primary composite outcome (ESKD or eGFR <15 mL/min/1.73 m^2^, sustained decrease of ≥40% in eGFR from baseline for ≥4 weeks, or kidney disease death) as compared with those assigned to placebo (HR=0.82 [95% CI=0.73–0.93]) [67]. FInerenone in reducinG cArdiovascular moRtality and mOrbidity in Diabetic Kidney Disease (FIGARO-DKD) [68] included kidney disease endpoints as secondary outcomes. Participants assigned to finerenone had a 23% lower risk of the composite kidney disease outcome of first occurrence of kidney failure, sustained decrease from baseline eGFR ≥57% for ≥4 weeks, or kidney disease death as compared to the placebo arm (HR=0.77 [95% CI: 0.60–0.99]) [68]. Both of these clinical trials included participants with T2D and DKD who were on a maximally tolerated dose of an ACE inhibitor or ARB [67, 68]. The FInerenone in chronic kiDney diseasE and type 2 diabetes: Combined FIDELIO-DKD and FIGARO-DKD Trial programme analYsis (FIDELITY) [57] prespecified meta-analysis reported that finerenone significantly reduced risk of kidney disease outcomes (kidney failure, sustained ≥57% decrease in eGFR, or kidney disease death) by 23% and the risk of cardiovascular endpoints (death from cardiovascular causes, nonfatal myocardial infarction, nonfatal stroke, or hospitalization for heart failure) by 14% versus placebo in >13,000 participants. Finerenone was well tolerated, but investigator-reported hyperkalemia (serum potassium concentration >5.5 mmol/l) was more common versus placebo (14.0% versus 6.9%, respectively) [57].

## Conclusions

DKD is a frequent and serious complication in people with T2D and diabetes is the most common cause of ESKD and kidney failure worldwide [59]. Glycemic control and blood pressure management, with preferential use of agents that attenuate the renin-angiotensin aldosterone system, have traditionally represented the cornerstone for prevention and treatment of DKD. Even though these measures may reduce albuminuria, their beneficial effects on GFR decline or progression to ESKD are modest [63, 64, 69, 70].

In recent studies, treatment with SGLT-2i and GLP-1 RA proved to reduce the risk for a combined major adverse cardiovascular event endpoint (including cardiovascular death, non-fatal myocardial infarction, or non-fatal stroke) [60, 71]. In the CREDENCE and the DAPA-CKD trials, treatment with canagliflozin and dapagliflozin were shown to reduce risks of substantial eGFR decline or kidney failure with a primary kidney disease outcome in adults with T2D who had DKD. These findings have inspired many organizations that produce clinical practice guidelines across the world to recommend these agents over other treatments in people with T2D and DKD and/or cardiovascular disease.

Despite these new therapeutic opportunities for treating people with T2D, the risk of DKD progression remains [11, 72]. There is evidence to support the role of the mineralocorticoid receptor through inflammation and fibrosis in the progression of DKD [72]. Treatment of DKD with older steroidal MRAs has not been widely implemented because of their high rate of unfavorable side effects such as hyperkalemia [72]. However, finerenone is a new non-steroidal MRA with less side effects and more potent anti-inflammatory and antifibrotic effects as compared with steroidal MRAs [73, 74]. Finerenone was shown to evoke kidney and cardiovascular protective effects in people with T2D and DKD [57, 67]. Therefore, promising new pharmacological drugs are available to be used in people with DKD.

Drugs like phosphodiesterase inhibitors, 5-hydroxytrytamine 2a receptor antagonists, aldosterone synthesis inhibitors, anti-inflammatory agents, and others are under clinical development. Such additional classes of agents might further increase the armamentarium in the treatment of DKD in the future [33, 75]. Even though new drugs will help to improve the prognosis of people with DKD, it becomes more and more a challenge for physicians to choose the most beneficial medication or combination of medications for an individual patient. There is a need to evaluate the kidney-protective effects of different treatment modalities based on individual characteristics. For example, it would be important to evaluate if different drugs might have a distinct efficacy in patients with DKD with and without albuminuria. Combination therapy with SGLT-2is and MRAs also need to be better explored to understand if benefits are additive. Additional clinical and real-world studies are warranted to elucidate best clinical practices.

It is important to emphasize the intention of this review, along with its limitations. We aimed to provide an overview on recent renal data of SGLT-2i, GLP-1 RAs, and MRAs. Most of the studies included in the review were cardiovascular outcome trials, with kidney outcomes as secondary outcomes. As such, they may not have sufficient power to provide confirmative answers on kidney-related endpoints, especially when examined by subgroups. Furthermore, for composite secondary kidney outcomes, examining each individual component of the composite outcome provided interesting information, but again, these results were underpowered to be considered confirmatory. With the composite renal outcomes of studies examining GLP-1 RAs driven primarily by reductions in albuminuria, the studies do not prove any beneficial effect of GLP-1 RA on kidney outcomes. Even though many of the results are not confirmatory, they are of interest to discuss potential effects in an exploratory sense. Results of these trials are thesis generating and should not be interpreted in a confirmatory sense. This highlights the need for future trials with kidney outcomes as primary outcomes of interest.

## Data Availability

Cited sources are available online

## Abbreviations

ACEi: Angiotensin-converting enzyme inhibitor
ADA: American Diabetes Association
AMPLITUDE-O: Effect of Efpeglenatide on Cardiovascular Outcomes
ARB: Angiotensin II receptor blocker
AWARD 7: Assessment of Weekly Administration of dulaglutide in Diabetes
BL: Baseline
CANVAS/CANVAS-R: Canagliflozin Cardiovascular Assessment Study
CI: Confidence interval
CKD: Chronic kidney disease
CKD-EPI: Chronic Kidney Disease Epidemiology Collaboration
CREDENCE: Canagliflozin and Renal Events in Diabetes with Established Nephropathy Clinical Evaluation
CVD: Cardiovascular disease
DAPA-CKD: Dapagliflozin and Prevention of Adverse Outcomes in Chronic Kidney Disease
DECLARE-TIMI 58: Dapagliflozin Effect on Cardiovascular Events-Thrombolysis in Myocardial Infarction 58
DKD: Diabetic kidney disease
eGFR: Estimated glomerular filtration rate
ELIXA: Evaluation of Lixisenatide in Acute Coronary Syndrome
EMPA-REG OUTCOME: Empagliflozin Cardiovascular Outcome Event Trial in Type 2 Diabetes Mellitus Patients
EMPEROR REDUCED: Empagliflozin Outcome Trial in Patients with Chronic Heart Failure, Reduced Ejection Fraction
ESKD: End-stage kidney disease
EXSCEL: Exenatide Study of Cardiovascular Event Lowering
FIDELIO-DKD: FInerenone in reducing kiDnEy faiLure and dIsease prOgression in Diabetic Kidney Disease
FIDELITY: FInerenone in chronic kiDney diseasE and type 2 diabetes: Combined FIDELIO-DKD and FIGARO-DKD Trial programme analysis
FIGARO-DKD: FInerenone in reducinG cArdiovascular moRtality and mOrbidity in Diabetic Kidney Disease
GFR: Glomerular filtration rate
GLP-1 RA: Glucagon-like peptide-1 receptor agonist
Harmony Outcomes: Effect of albiglutide, when added to standard blood glucose lowering therapies, on major cardiovascular events in subjects with type 2 diabetes
HbA1c: Glycated hemoglobin
HR: Hazard ratio
KDIGO: Kidney Disease Improving Global Outcomes
LDL: Low density lipoprotein
LEADER: Liraglutide Effect and Action in Diabetes: Evaluation of Cardiovascular Outcome Results
LS: Least squares
LSM: Least square method
MDRD: Modification of Diet in Renal Disease
MRA: Mineralocorticoid receptor antagonist
ns: Non-significant
NYHA: New York Heart Association
RAS: Renin-angiotensin system
REWIND: Dulaglutide and Cardiovascular Outcomes in Type 2 Diabetes
SGLT-2i: Sodium glucose transport protein 2 inhibitor
SGLT2: Sodium glucose transport protein 2
SUSTAIN-6: Trial to Evaluate Cardiovascular and Other Long-term Outcomes with Semaglutide in Subjects with Type 2 Diabetes
T2D: Type 2 diabetes
UACR: Urinary albumin to creatinine ratio
VERTIS- CV: EValuation of ERTugliflozin effIcacy and safety – CardioVascular outcomes
yrs: Years
